# Boosting Throughput and Efficiency of Hardware Spiking Neural Accelerators Using Time Compression Supporting Multiple Spike Codes

**DOI:** 10.3389/fnins.2020.00104

**Published:** 2020-02-14

**Authors:** Changqing Xu, Wenrui Zhang, Yu Liu, Peng Li

**Affiliations:** ^1^School of Microelectronics, Xidian University, Xi'an, China; ^2^Department of Electrical and Computer Engineering, University of California, Santa Barbara, Santa Barbara, CA, United States; ^3^Department of Electrical and Computer Engineering, Texas A&M University, College Station, TX, United States

**Keywords:** time compression, spiking neural networks, input-output-weighted spiking neurons, time averaging, liquid-state machine

## Abstract

Spiking neural networks (SNNs) are the third generation of neural networks and can explore both rate and temporal coding for energy-efficient event-driven computation. However, the decision accuracy of existing SNN designs is contingent upon processing a large number of spikes over a long period. Nevertheless, the switching power of SNN hardware accelerators is proportional to the number of spikes processed while the length of spike trains limits throughput and static power efficiency. This paper presents the first study on developing temporal compression to significantly boost throughput and reduce energy dissipation of digital hardware SNN accelerators while being applicable to multiple spike codes. The proposed compression architectures consist of low-cost input spike compression units, novel input-and-output-weighted spiking neurons, and reconfigurable time constant scaling to support large and flexible time compression ratios. Our compression architectures can be transparently applied to any given pre-designed SNNs employing either rate or temporal codes while incurring minimal modification of the neural models, learning algorithms, and hardware design. Using spiking speech and image recognition datasets, we demonstrate the feasibility of supporting large time compression ratios of up to 16×, delivering up to 15.93×, 13.88×, and 86.21× improvements in throughput, energy dissipation, the tradeoffs between hardware area, runtime, energy, and classification accuracy, respectively based on different spike codes on a Xilinx Zynq-7000 FPGA. These results are achieved while incurring little extra hardware overhead.

## 1. Introduction

Spiking neural networks (SNNs) closely emulate the spiking behaviors of biological brains (Ponulak and Kasinski, 2011). Moreover, the event-driven nature of SNNs offer potentials in achieving great computational/energy efficiency on hardware neuromorphic computing systems (Furber et al., 2014; Merolla et al., 2014). For instance, processing a single spike may only consume a few pJ of energy on recent neuromorphic chips such as IBM's TrueNorth (Merolla et al., 2014) and Intel's Loihi (Davies et al., 2018).

SNNs support various rate/temporal spike codes among which rate coding using Poisson spike trains is popular. However, in that case, the low-power advantage of SNNs may be offset by long latency during which many spikes are processed for ensuring decision accuracy (Kim et al., 2018; Park et al., 2019). Various temporal codes have been attempted to improve the efficiency of information representation (Thorpe, 1990; Thorpe et al., 2001; Izhikevich, 2002; Kayser et al., 2009; Kim et al., 2018). The time-to-first-spike coding encodes information using arrival time of the first spike (Thorpe et al., 2001). Phase coding (Kayser et al., 2009) encodes information in a spike by its phase relative to a periodic reference signal (Kim et al., 2018). For example, Kim et al. (2018) converts a pre-trained ANN to an approximate SNN by exploring a phase coding method to encode input spikes by the phase of a global reference clock and achieves latency reduction over the rate coding for image recognition.Other studied coding schemes include rank-order coding (Thorpe, 1990) and resonant burst coding (Izhikevich, 2002). While the on-going neural coding work shows promises, no coding is considered universally optimal thus far. The achievable latency/spike reduction of a particular code can vary widely with network structure and application. Furthermore, software/hardware overheads of various codes are yet to be fully evaluated.

Except for studying on various codes to attempt to improve the efficiency of information representation, there are some researches utilizing neural adaptation to achieves a high coding efficiency. For example, Bohte (2012) proposed a multiplicative Adaptive Spike Response Model which can achieve a high coding efficiency and maintain the coding efficiency over changes in the dynamic signal range of several orders of magnitude. In Zambrano and Bohte (2016) and Zambrano et al. (2017), author proposed an Adapting Spiking Neural Network (ASNN) based on adaptive spiking neurons which can use an order of magnitude fewer spikes to get a good performance. In Zambrano et al. (2019) and O'Connor et al. (2017), they use the speed of adaptation and the effective spike height to control the precision of the spike-based neural coding. By utilizing neural adaptation, fire rate can be reduced, effectively, which saves a large amount of energy. Due to the fact that large numbers of neurons fire in irregular bursts (Trappenberg, 2009), a spike traffic compression technique is proposed to reduce traffic overhead and improving throughput on the Network-on-Chip based Spiking neural Network (Carrillo et al., 2012) The proposed compression technique can compress spike events generated by different neural cells within the same neuron facility into a single packet.

**Figure 1:**
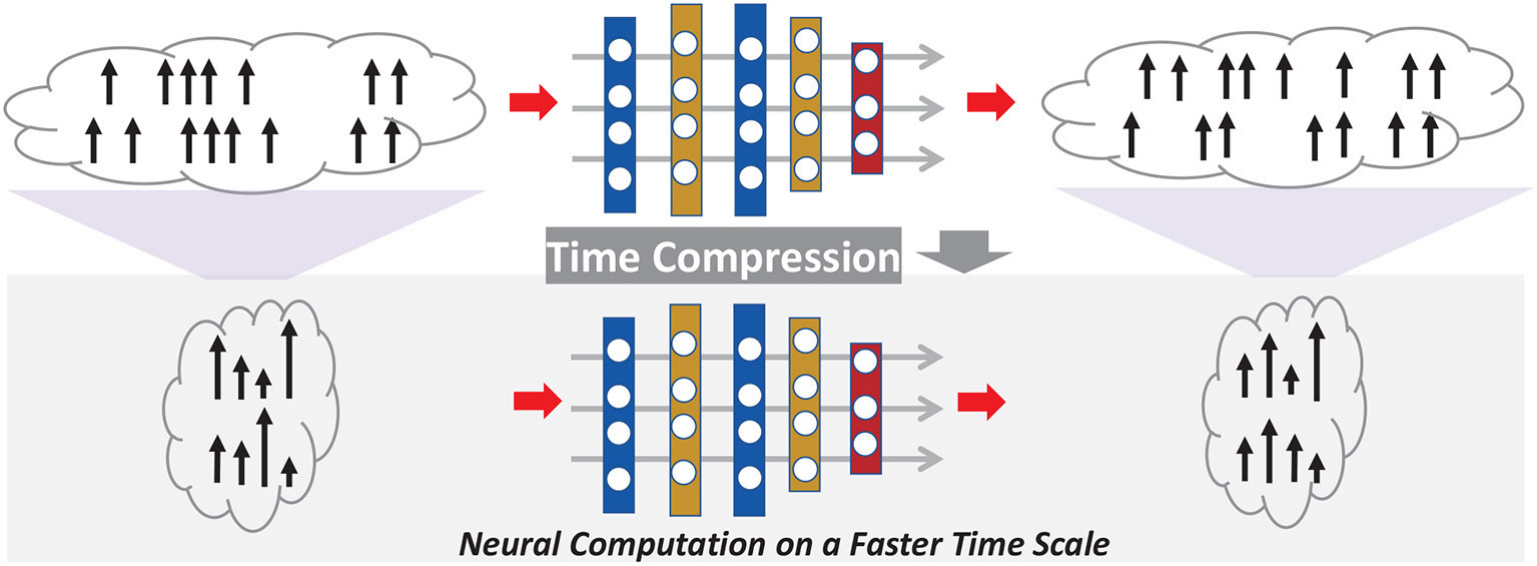
Proposed general time compression for SNNs.

Rather than advocating a particular code, *for the first time*, we focus on an orthogonal problem: temporal compression applicable to any given SNN (accelerator) and spike code to boost throughput and energy efficiency. We propose a general compression technique that preserves both the spike count and temporal characteristics of the original SNN with low information loss, as shown in Figure 1. Unlike the work in Zambrano and Bohte (2016), Zambrano et al. (2017), and Carrillo et al. (2012), our work transparently compresses the duration of the spike trains, hence classification latency, on top of an existing rate/temporal code. More broadly, this work extends the notion of weight/model pruning/compression of DNN accelerators from the spatial domain to the temporal domain. The proposed technique does not alter the given code already put in place; it intends to further reduce latency via time compression.

The contributions of this paper include: (1) the first general time-compression technique transparently compressing spike train duration of a given SNN and achieving large latency reduction on top of the spike codes that come with the SNN, (2) facilitating the proposed time compression by four key ideas: spike train compression using a weighted representation, a new family of input-output-weighted (IOW) spiking neural models for processing time-compressed spike trains for multiple spike codes, scaling of time constants defining neural, synaptic, and learning dynamics, and low-cost support of flexible compression ratios (powers of two or not) using time averaging, (3) low-overhead hardware modifications of a given SNN accelerator to operate it on a compressed time scale while preserving the spike counts and temporal behaviors in inference and training, (4) a time-compressed SNN (TC-SNN) accelerator architecture and its programmable variant (PTC-SNN) operating on a wide range of (programmable) compression ratios and achieving significantly improved latency, energy efficiency, and tradeoffs between latency/energy/classification accuracy.

We demonstrate the proposed TC-SNN and PTC-SNN compression architectures by realizing several liquid-state machine (LSM) spiking neural accelerators with a time compression ratio up to 16:1 on a Xilinx Zynq-7000 FPGA. Using the TI46 Speech Corpus (Liberman et al., 1991), the CityScape image recognition dataset (Cordts et al., 2016), and N-TIDIGITS18 dataset (Anumula et al., 2018), we demonstrate the feasibility of supporting large time compression ratios of up to 16×, delivering up to 15.93×, 13.88×, and 86.21× improvements in throughput, energy dissipation, the tradeoffs between hardware area, runtime, energy, and classification accuracy, respectively based on various spike coding mechanisms including burst coding (Park et al., 2019) on a Xilinx Zynq-7000 FPGA. These results are achieved while incurring little extra hardware overhead.

## 2. Materials and Methods

### 2.1. Proposed Time-Compressed Neural Computation

This work aims to enable time-compressed neural computation that preserves the spike counts and temporal behaviors in inference and training of a given SNN while significantly improving latency, energy efficiency, and tradeoffs between latency/energy/classification accuracy. We develop four techniques for this objective: (1) spike train compression using a weighted representation, (2) a new family of input-output-weighted (IOW) spiking neural models processing time-compressed spike trains for multiple spike codes, (3) scaling of time constants of neural, synaptic, and learning dynamics, and (4) low-cost support of flexible compression ratios (powers of two or not) using time averaging.

#### 2.1.1. Spike Train Compression in Weighted Form

**Figure 2:**
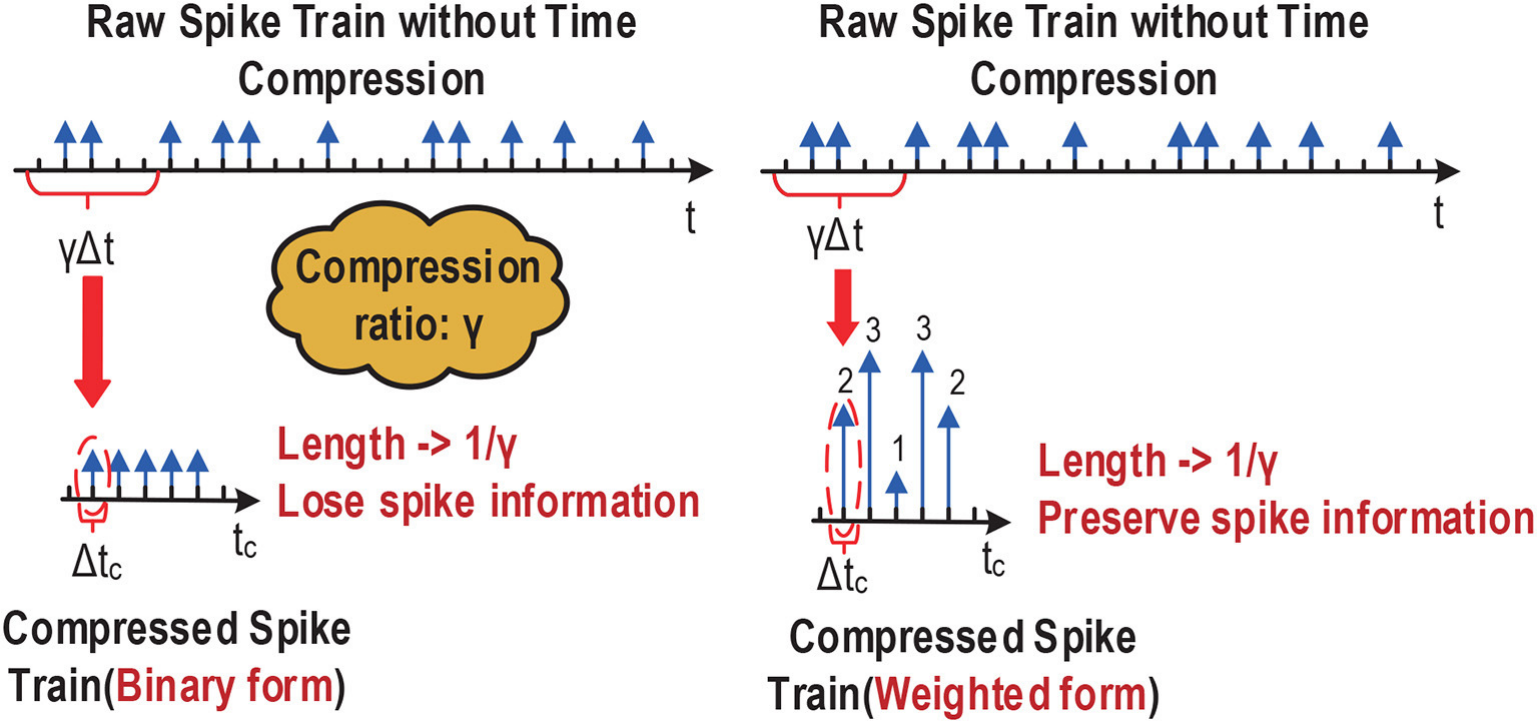
Binary vs. (compressed) weighted spike trains.

We time-compress a given spiking neural network first by shrinking the duration of the input spike trains. To support large compression ratios hence significant latency reductions, we represent the compressed input trains using an weighted form. Typical binary spike trains with temporal sparsity may be time-compressed into another binary spike train of a shorter duration. However, as shown in Figure 2, the spike count and temporal characteristics of the uncompressed train can only be preserved under a small compression ratio bound by the minimal interspike interval. More aggressive compression would lead to merging multiple adjacent spikes into a single spike, resulting in significant alterations of firing count and temporally coded information. This severely limits the amount of compression possible. Instead, we propose a new weighted form for representing compressed spike trains, where multiple adjacent binary spikes are compressed into a single weighted spike with a weight value equal to the number of binary spikes combined, allowing preservation of spike information even under very large compression ratios (Figure 2). Compared to the uncompressed spike train, the compressed spike train preserved the information of spike count and its temporal resolution drops to 1/γ, where γ is the compression ratio.

#### 2.1.2. Input-Output-Weighted (IOW) Spiking Neurons

**Figure 3:**
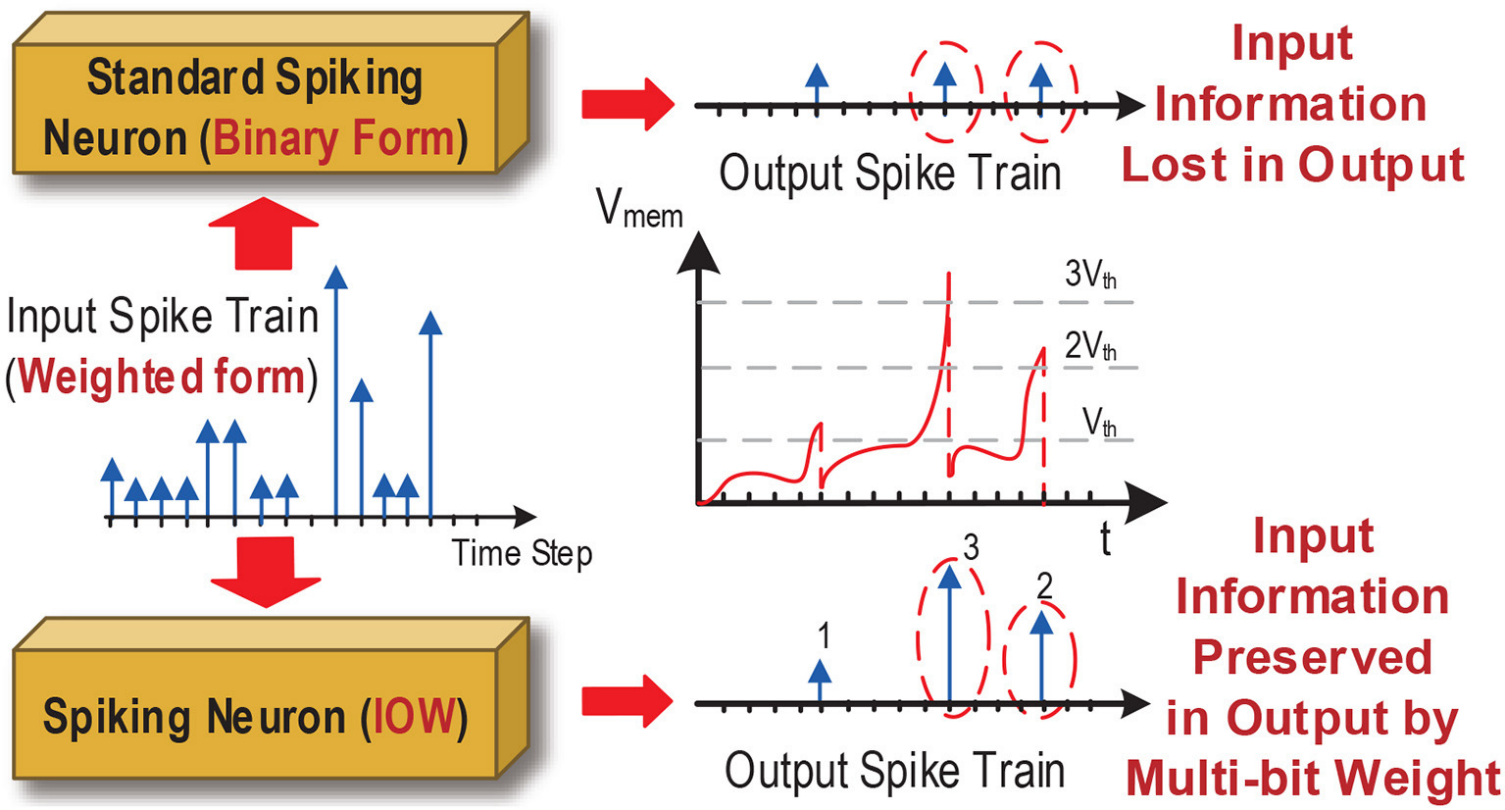
Binary vs. weighted output spikes.

As such, each spiking neuron would process the received input spike trains in the weighted form. Furthermore, as shown in Figure 3, under large compression ratios the membrane potential of a spiking neuron may rise high above the firing threshold voltage within a single time step as a result of receiving input spikes with large weights. In this case, outputting spike trains in the standard binary form can lead to significant loss of input information, translating into large performance loss as we demonstrate in our experimental results. Instead, we propose a new family of input-output-weighted (IOW) spiking neural models which take the input spike trains in the weighted form and produce the output spike train in the same weighted form, where the multi-bit weight value of each output spike reflects the amplitude of the membrane potential as a multiple of the firing threshold. Spiking neuronal models such as the leaky integrate-and-fire (LIF) model and other models supporting various spike codes can be converted to their IOW counterpart with streamlined low-overhead modification as detailed later.

#### 2.1.3. Scaling of Time Constants of SNN Dynamics

The proposed compression is general in the sense that it intends to preserve the spike counts and temporal behaviors in the neural dynamics, synaptic responses, and dynamics employed in the given SNN such that no substantial alterations are introduced by compression other than that the time-compressed SNN just effectively operates on a faster time scale. The dynamics of the cell membrane is typically specified by a membrane time constant τ_*m*_, which controls the process of action potential (spike) generation and influences the information processing of each spiking neuron (Gerstner and Kistler, 2002). Synaptic models also play an important role in an SNN and may be specified by one or multiple time constants, translating received spike inputs into a continuous synaptic current waveform based on the dynamics of a particular order (Gerstner and Kistler, 2002). Finally, Spike traces or temporal variables filtered with a specific time constant may be used to implement spike-dependent learning rules (Thorpe et al., 2001; Zhang et al., 2015).

**Figure 4:**
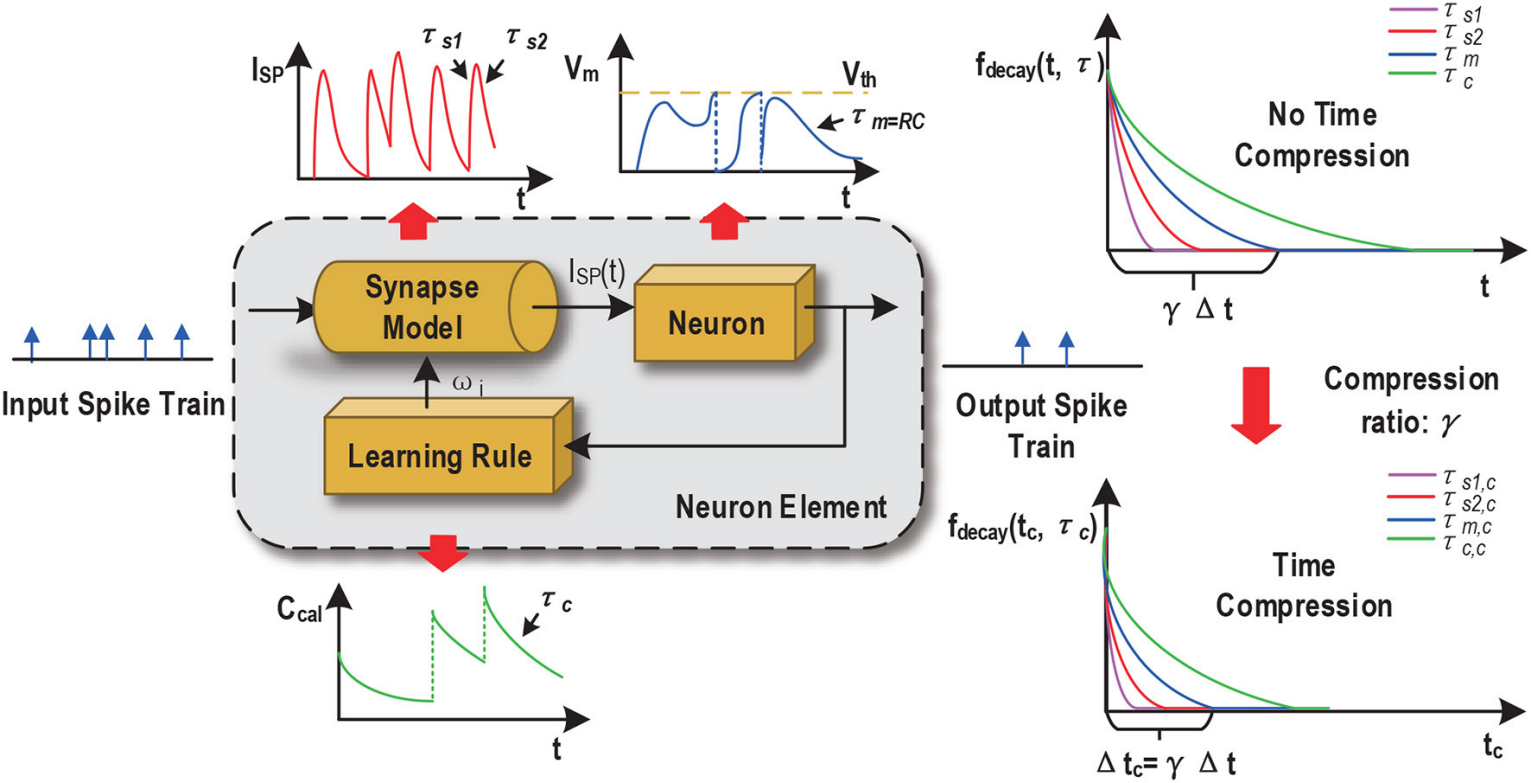
Scaling of time constants of SNN dynamics.

Maintaining the key spiking/temporal characteristics in the neural, synaptic, and learning processes is favorable because: (1) the SNNs with time compression essentially attains pretty much the same dynamic behavior like before such that the classification performance would be also similar to the one under no time compression, i.e., no large performance degradation is expected when employing time compression; (2) the deployed learning rules need no modification and the same rules can effectively train the SNNs with time compression. Spike-dependent training algorithms often make use of internal dynamics. For example, the probabilistic spike-dependent learning rule (Zhang et al., 2015) uses a first-order calcium dynamics to characterize the time-averaged output firing rate. Attaining the above goal entails proper scaling of the time constants associated with these processes as a function of the time compression ratio as shown in Figure 4.

Without loss of generality, consider a decaying first order dynamics *ẋ*(*t*) = −*x*(*t*)/τ with time constant τ. For digital hardware implementation, forward Euler discretization may be adopted to discretize the dynamics over time:

(1) $$\begin{array}{l} \begin{array}{c} X\left(t+\Delta t\right)=X\left(t\right)\left(1-\frac{\Delta t}{\tau }\right)=X\left(t\right)\left(1-\frac{1}{\tau_{nom}}\right) \end{array} \end{array}$$

where Δ*t* is the discretization time stepsize and τ_*nom*_ = τ/Δ*t* is the normalized time constant used in digital hardware implementation. Now denote the target time compression ratio by γ (γ ≥ 1). The discretization stepsize with time compression is: Δ*t*_*c*_ = γ Δ*t*, i.e., one time step of the time-compressed SNN equals to γ time steps of the uncompressed SNN. Based on (1), discretizing the first order dynamics with time compression for one step gives:

(2) $$\begin{array}{l} X\left(t+\Delta t_{c}\right)=X\left(t\right)\left(1-\frac{1}{\tau_{nom,c}}\right)=X\left(t\right){\left(1-\frac{1}{\tau_{nom}}\right)}^{\gamma }, \end{array}$$

where τ_*nom, c*_ is the normalized time constant with compression. Linearly scaling τ_*nom, c*_ by $\tau_{nom,c}=\frac{\tau_{nom}}{\gamma }$ is equivalent to: $X\left(t+\Delta t_{c}\right)\approx X\left(t\right)\left(1-\frac{1}{\tau_{nom}/\gamma }\right)$, which produces large errors when γ ≫ 1. Instead, we get an accurate τ_*nom, c*_ value according to: $\tau_{nom,c}\;=\;\frac{1}{1-{\left(1-\frac{1}{\tau_{nom}}\right)}^{\gamma }}$.

#### 2.1.4. Flexible Compression Ratios Using Time Averaging

Digital multipliers and dividers are costly in area and power dissipation. Normalized time constants in a digital SNN hardware accelerator are typically set to a power of 2, i.e., $\tau_{nom}=2^{K}$ such that the dynamics can be efficiently implemented by a shifter rather than expensive multipliers and dividers (Zhang et al., 2015). However, it may be desirable to choose a compression ratio and/or scale each time constant continuously in a wide integer range, e.g., within {1, 2, 3, …, 16}. In this case, each scaled normalized time constant τ_*nom, c*_ may not be a power of 2. For example, when τ_*nom, c*_ = 10, τ_*nom, c*_ is far away from its two nearest powers of 2, namely 8 and 16. Setting τ_*nom, c*_ to either of the two would lead to large errors.

**Figure 5:**
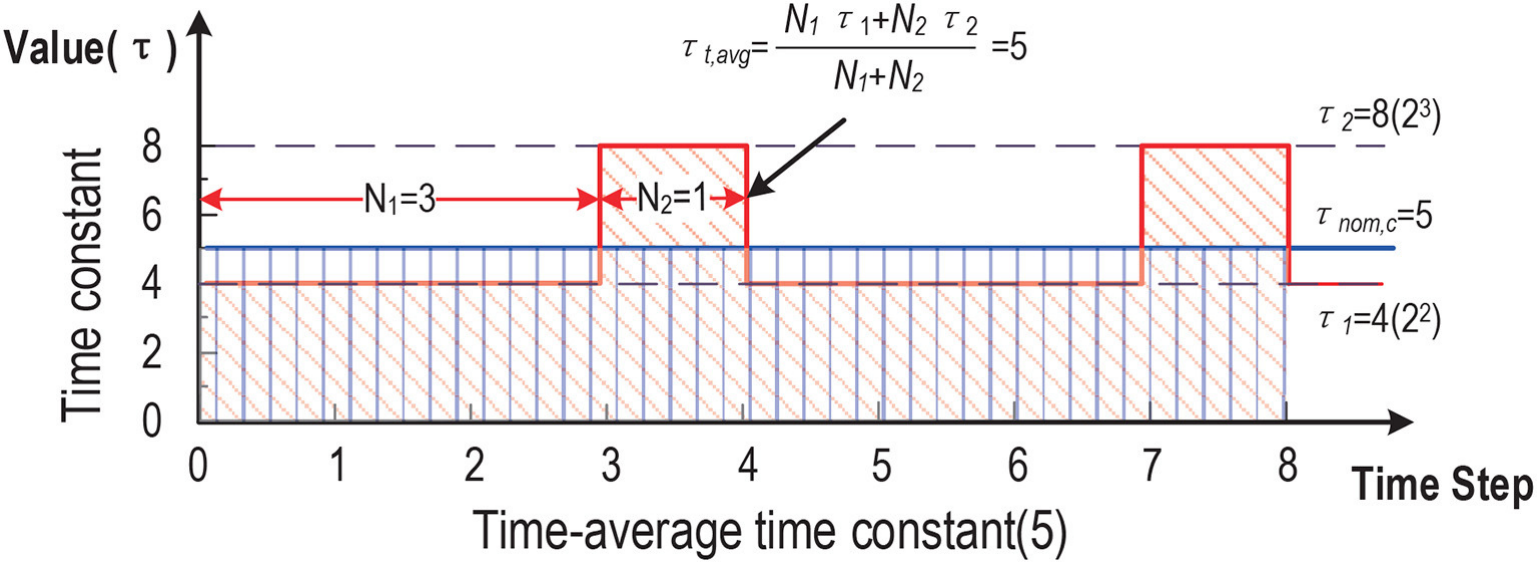
Time-averaged time constants: the realized averaged time constant is 5.

We propose a novel time averaging approach to address the above problem (Figure 5). For a given scaled normalized τ_*nom, c*_, we find its two adjacent powers of 2: $2^{K_{2}}\leq \tau_{nom,c}\leq 2^{K_{1}}$. We decay the targeted first order dynamics by toggling its scaled normalized time constant between two values: $2^{K_{2}}$ and $2^{K_{1}}$. Since each of them is a power of two, the corresponding decaying behavior can be efficiently realized using a shifter. The usage frequencies of $2^{K_{2}}$ and $2^{K_{1}}$ are properly chosen such the time-averaged time constant is equal to the desired τ_*nom, c*_. Figure 5 shows how the time-averaged (normalized) time constant value of 5 is achieved by averaging between two compression ratios 4 and 8.

### 2.2. Proposed Input-and-Output Weighted (IOW) Spiking Neural Models

Any given spiking neural model can be converted into its input-and-output (IOW) counterpart based on straightforward low-overhead modifications. Without loss of generality, we consider conversion of two models: the standard leaky integrate-and-fire (LIF) neuron model, which has been widely used in many SNNs including ones based on rating coding, and one of its variants for supporting burst coding. The same approach can be taken to convert other types of neuron models.

#### 2.2.1. IOW Neurons Based on Standard LIF Model

The LIF model dynamics is Gerstner and Kistler (2002):

(3) $$\begin{array}{l} \tau_{m}\frac{du}{dt}=-u\left(t\right)+RI\left(t\right), \end{array}$$

where *u*(*t*) is the membrane potential, τ_*m*_ = *RC* is the membrane time constant, and *I*(*t*) is the total received post-synaptic current given by:

(4) $$\begin{array}{l} I\left(t\right)=\underset{i}{\sum }w_{i}\underset{f}{\sum }\alpha \left(t-t_{i}^{\left(f\right)}\right), \end{array}$$

where *w*_*i*_ is the synaptic weight from the pre-synaptic neuron *i*, $\alpha \left(t\right)=\frac{q}{\tau_{s}}\exp\left(-\frac{t}{\tau_{s}}\right)H\left(t\right)$ for a first order synaptic model with time constant τ_*s*_, *H*(*t*) is the Heaviside step function, and *q* is the total charge injected into the post-synaptic neuron through a synapse of a weight of 1.

Once the membrane potential reaches the firing threshold *u*_*th*_, an output spike is generated and the membrane potential is reset according to:

(5) $$\begin{array}{l} \underset{\delta ->0;\delta >0}{\lim}u\left(t^{\left(f\right)}+\delta \right)=u\left(t^{\left(f\right)}\right)-u_{th}, \end{array}$$

where *t*^(*f*)^ is the firing time.

IOW LIF neurons shall process weighted input spikes because of time compression with the modified synaptic input:

(6) $$\begin{array}{l} I\left(t\right)=\underset{i}{\sum }w_{i}\underset{f}{\sum }\omega_{spike,i}^{f}\alpha \left(t-t_{i}^{\left(f\right)}\right), \end{array}$$

where a weight $\omega_{spike,i}^{f}$ is introduced for each input spike.

IOW LIF neurons shall also generate weighted output spikes. According to Figure 3, we introduce a set of firing thresholds {*u*_*th*_, 2*u*_*th*_,…,n*u*_*th*_} with each being a multiple of the original threshold *u*_*th*_. At each time step *t*, an output spike is generated whenever the membrane potential reaches above any firing threshold from the set and the weight of the output spike is determined by the actual threshold crossed. For example, when *ku*_*th*_ ≤ *u*(*t*) < (*k* + 1)*u*_*th*_, the output spike weight is set to *k*. Upon firing, the membrane potential is reset according to:

(7) $$\begin{array}{l} \underset{\delta ->0;\delta >0}{\lim\;u(t^{\left(f\right)}+\delta )}=\{\begin{array}{cc} u\left(t^{\left(f\right)}\right)-u_{th,} & u_{th}\leq u\left(t^{\left(f\right)}\right)<2u_{th}\\ u\left(t^{\left(f\right)}\right)-u2_{th,} & 2u_{th}\leq u\left(t^{\left(f\right)}\right)<3u_{th}\\ \ldots & \ldots \\ u\left(t^{\left(f\right)}\right)-nu_{th,} & u\left(t^{\left(f\right)}\right)\geq nu_{th} \end{array} \end{array}$$

#### 2.2.2. IOW Neurons Based on Bursting LIF Model

The LIF model for burst coding is also based on (3) (Park et al., 2019). A bursting function *g*_*i*_(*t*) is introduced to implement the bursting behavior per each presynaptic neuron *i* (Park et al., 2019):

(8) $$\begin{array}{l} g_{i}(t)=\{\begin{array}{ll} \beta g_{i}(t-\Delta t), & \text{if }E_{i}(t-\Delta t)=1\\ 1, & \text{otherwise} \end{array} \end{array}$$

where β is a burst constant, *E*_*i*_(*t* − Δ*t*) = 1 if the presynaptic neuron *i* fired at the previous time step and otherwise *E*_*i*_(*t* − Δ*t*) = 0. We assume a zero-th order synaptic response model. Per input spikes from the presynaptic neuron *i*, the firing threshold voltage is modified from *u*_*th*_ to *g*_*i*_(*t*)*u*_*th*_ and the corresponding reset characteristic of the membrane potential after firing is:

(9) $$\begin{array}{l} \underset{\delta ->0;\delta >0}{\lim}u\left(t^{\left(f\right)}+\delta \right)=u\left(t^{\left(f\right)}\right)-g_{i}\left(t^{\left(f\right)}\right)u_{th}. \end{array}$$

Furthermore, the total post-synaptic current is:

(10) $$\begin{array}{l} I\left(t\right)=\underset{i}{\sum }w_{i}\underset{f}{\sum }g_{i}\left(t\right)\alpha \left(t-t_{i}^{\left(f\right)}\right). \end{array}$$

To implement the IOW version of the LIF model with burst coding, we modify the burst function to:

(11) $$\begin{array}{l} g_{i}(t)=\{\begin{array}{ll} \beta^{\omega_{spike,i}(t)}g(t-\Delta t), & \text{if }E_{i}(t-\Delta t)=1\\ 1, & \text{otherwise} \end{array} \end{array}$$

Similar to the case of the IOW LIF model, we use a set of firing thresholds to determine the weight of each output spike and a behavior similar to (7) for reset. The only difference here is that the adopted set of firing thresholds are *g*_*i*_(*t*)*u*_*th*_, 2*g*_*i*_(*t*)*u*_*th*_, ⋯ ,*ng*_*i*_(*t*)*u*_*th*_.

### 2.3. Time-Compressed SNN Accelerator Architectures

**Figure 6:**
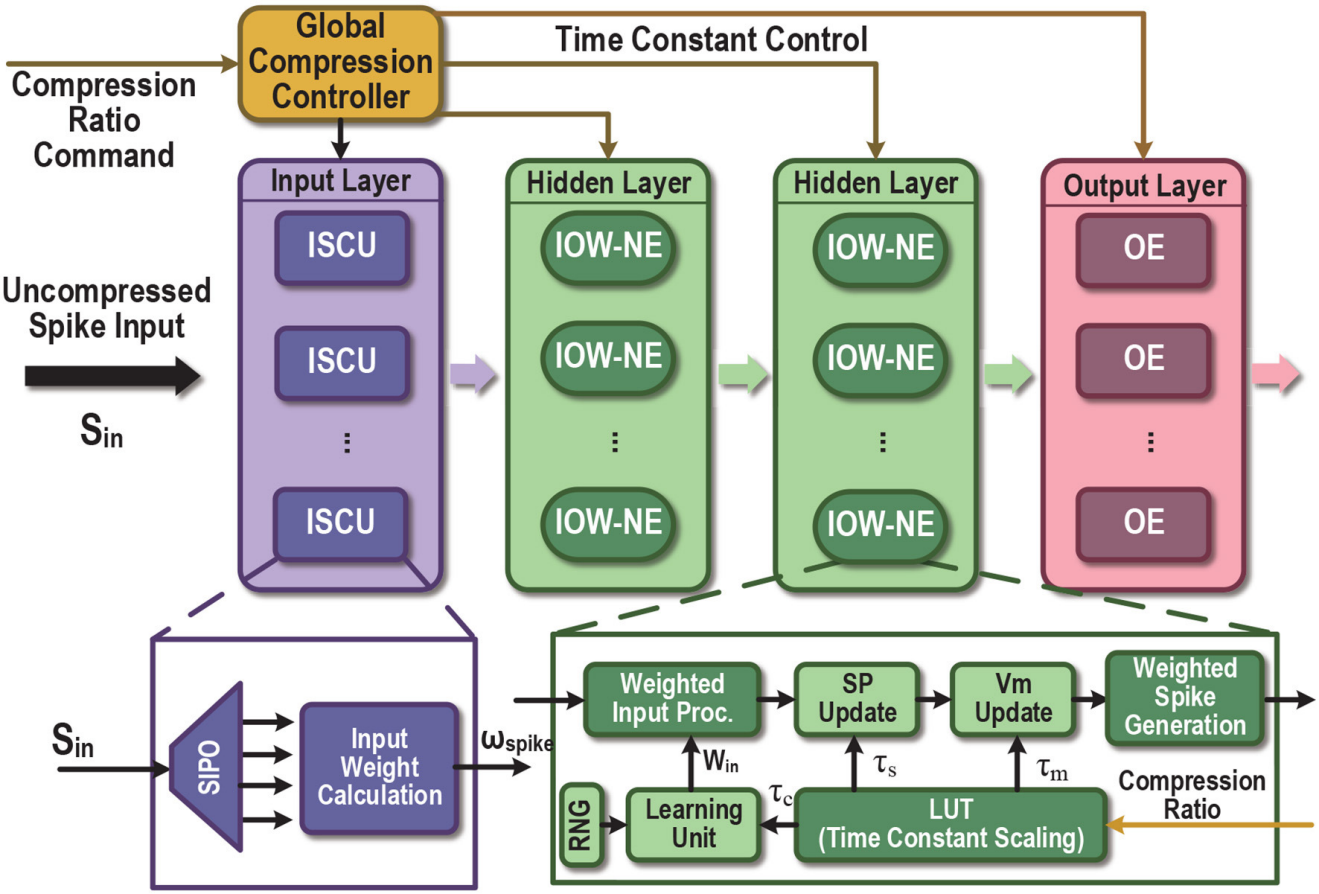
Proposed time-compressed SNN architecture with programmable compression ratio (PTC-SNN). ISCU, input spike compression unit; SIPO, serial-in and parallel-out; IOW-NE, input-output-weighted spiking neuron element; SP, synapse response; NE, regular binary-input-output neuron element; Vm, membrane potential. The LUT enables programmable scaling of time constants of the neuron/synaptic models and the learning unit.

The proposed time compression technique can be employed to support a fixed time compression ratio or user-programmable time compression ratio, leading to the time-compressed SNN (TC-SNN) and programmable time-compressed SNN (PTC-SNN) architectures, respectively. We describe the more general PTC-SNN architecture shown in Figure 6. It can be adopted for any pre-designed SNN hardware accelerator for added programmable time compression. PTC-SNN introduces three streamlined additions and minor modifications to the embedded SNN accelerator to enable application and coding independent time compression.

**Figure 7:**
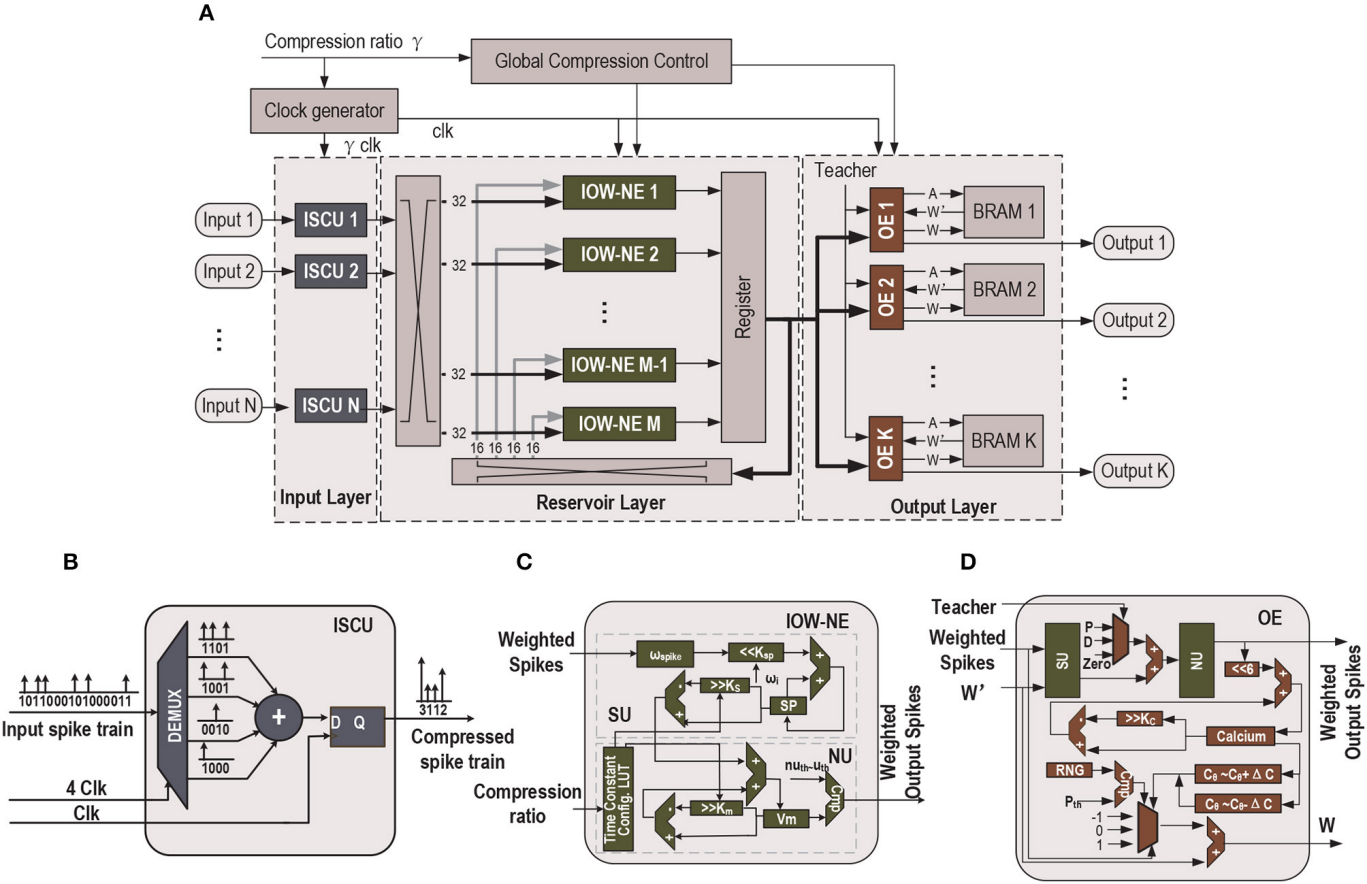
**(A)** Top-level block diagram of the proposed time-compressed liquid state machine (LSM) SNN accelerator with programmable compression ratio (PTC-SNN), **(B)** ISCU with 4:1 time compression, **(C)** LIF IOW neuron: SU - synaptic unit, NU - neural unit, and **(D)** OE: P - Potentiation, D - Depression (Wang et al., 2016).

For demonstration purpose, we show how an existing liquid state machine (LSM) SNN accelerator (Wang et al., 2016) can be re-designed to a TC-SNN and PTC-SNN. As shown in Figure 7A, the architecture consists of an input layer, a reservoir layer and an output layer. In the input layer, a set of input-spike compression units (ISCUs), one for each input spike channel, are used to convert the raw binary input spike trains into the more compact weighted form with shortened time duration. A user-specified command sets the time compression ratio of all ISCUs through the Global Compression Controller. ISCUs compress the given spike channels without assuming sparsity of the input spike trains and can support large compression ratios. In the reservoir layer, we introduce modest added hardware overhead to replace all original silicon spiking neurons by their input-output-weighted neuron elements (IOW-NEs) counterparts which is shown in Figure 7C. The output layer is composed of output elements (OEs) and all the OEs update the corresponding synaptic weights in parallel. These plastic synaptic weights are stored in the corresponding block RAMs (BRAMs). The external input spikes are sent to their target IOW-NEs through a crossbar switching interface. The spikes generated by the IOW-NEs are buffered in a wide register. Then, the spikes in the register are sent to other IOW-NEs through another crossbar switching interface. At the same time, the spikes in the register are also sent to each OE in the output layer as the reservoir response. We apply a biologically plausible supervised learning rule, which is proposed in Zhang et al. (2015), to realize supervised learning, a teacher signal is used to modulate the firing activity of each OE and implement a particular form of Hebbian learning. For the recognition phase, if the fire count of the OE corresponding to a sample's true class is the most, this particular speech sample is successfully recognized. Because the output spikes have weight, we use the sum of spike multiply by spike weight as fire count. Finally, all time constants in the SNN are scaled by the Global Compression Controller according to a user-specified compression ratio command.

**[Input Spike Compression Unit (ISCU)]** Each input spike channel is compressed by one low-cost ISCU according to the user-specified compression ratio γ. When each uncompressed spike input channel is fed by a single binary serial input, a demultiplexer is utilized in the ISCU to perform the reconfigurable serial-in and parallel-out (SIPO) operation to convert the serial input into γ parallel outputs, as shown in Figure 7B. If the input spike channel is supplied by parallel spike data, the SIPO operation is skipped. In order to achieve real-time input spike compression, the work frequency of the ISCU is γ times that of reservoir layer and output layer. During each clock cycle, the γ bits of the parallel outputs are added by an adder, which effectively combines these spikes into a single weighted spike with a weight value set by the output of the adder. No spike count loss is resulted as the sum of spike weights is same as the total number of binary spikes in the raw spike input train. The global temporal spike distribution of the input spike train is preserved up to the temporal resolution of the compressed spike train. As shown in Figure 7B, when compression ratio is 4:1, the first four serial input “1100” is converted to a parallel form. The four parallel spikes "1100" are added by an adder and converted into a single spike with weight “2”.

There is unavoidable loss of fine temporal resolution since γ adjacent spikes in the raw input spike train are combined into a single weighted spike. When the compression ratio is low, this loss of temporal resolution may be negligible while large latency and energy reduction can be achieved. As will be demonstrated by our experimental studies, it is possible to explore aggressively large input compression ratios, e.g., 16:1, for huge latency and energy dissipation improvements. Under this case, it is still possible to retain a decent classification performance as the lost temporal resolution can be partially compensated by training, which operates under the same time compression ratio.

**[Input-Output-Weighted (IOW) Neuron Elements]** We discuss efficient hardware realization of the IOW spiking neural models (section 2.2). The IOW neuron element (IOW-NE) is shown in Figure 7C, which consists of a synaptic unit (SU), a neural unit (NU), and a time constant configuration module, described later. SU realizes a discretized version of (6). As in many practical implementations of hardware SNNs, each ω_*i*_ is constrained to be in the form of 2^*K*^. The product of ω_*spike, i*_ · ω_*i*_ is efficiently realized by left shifting ω_*spike, ki*_ by K bits. NU performs membrane potential *u*(*t*) update based on discretization of (3) and reset behavior (7). NU generates a weighted output spike when *u*(*t*) is above certain threshold in the firing threshold set *u*_*th*_, 2*u*_*th*_, ⋯ .

The design of IOW LIF neurons with burst coding is almost identical to that of the IOW LIF neurons except for the following differences. We add a LUT to store the set of firing thresholds {*g*_*i*_(*t*)*u*_*th*_, 2*g*_*i*_(*t*)*u*_*th*_, ⋯ }, which are calculated based on (11). Because *g*_*i*_(*t*)*u*_*th*_ might not be in the form of 2^*K*^, a multiplier is used to compute the product *g*(*t*) · *u*_*th*_ · ω_*i*_ · ω_*spike, i*_.

**[Output Elements (OE)]** In output elements (OE), we apply the a biologically plausible supervised learning rule which is proposed in Zhang et al. (2015). The similar functional blocks (SU, NU) are used to calculate the state variables, and its implementation is the same as the blocks in Figure 7C, except that the internal fixed synaptic weight is replaced by a plastic synaptic weight. The plastic synaptic weights are stored in a BRAM updated by the biologically plausible learning rule (Zhang et al., 2015). In the learning rule, the Calcium concentration C is calculated by

(12) $$\begin{array}{l} C\left(t\right)=C\left(t-1\right)-\frac{C\left(t-1\right)}{\tau_{c}}+E\left(t\right) \end{array}$$

where E(t) is the spiking event at current time step and τ_*c*_ is the time constant of calcium concentration. In our design, τ_*c*_ is in the form of $2^{K_{c}}$. The weight of synapse between current output neuron and the i-th reservoir neuron *w*_*i*_ is updated by

(13) $$\begin{array}{l} \{\begin{array}{c} w_{i}=w_{i}+\Delta w\text{ with }P\text{ if }C_{\theta }<C<(C_{\theta }+\Delta C)\\ w_{i}=w_{i}^{\prime }-\Delta w\text{ with }P\text{ if }(C_{\theta }-\Delta C)<C<C_{\theta } \end{array} \end{array}$$

where *P*, *C*_θ_ and Δ*C* are the update probability, the Calcium concentration threshold and margin width, respectively. In our design, *P* is 2%, *C*_θ_ is 320 and Δ*C* is 192. According to Figure 7D, besides the components for Vmem updating, each OE involves additional logic to realize (12) and (13). Once a synaptic weight is updated, it is written back to the BRAM. The update probability in (13) is simply realized by a comparator and a random number generator (RNG). To realize the spike-based supervised learning rule, the teacher signal is used to add an additional injection into each readout neuron to modulate the firing activity of each OE.

## 3. Results

The proposed time-compressed SNN (TC-SNN) architecture with a fixed compression ratio and the more general programmable PTC-SNN architecture with user-programmable compression ratio can be adopted to re-design any given digital SNN accelerator to a time-compressed SNN accelerator with low additional design overhead in a highly streamlined manner. For demonstration purpose, we show how an existing liquid state machine (LSM) SNN accelerator can be re-designed to a TC-SNN and PTC-SNN on a Xilinx Zynq-7000 FPGA. The LSM is a recurrent spiking neural network model. With its spatio-temporal computing power, it has demonstrated promising performances for various applications (Maass et al., 2002). Based on the design of the Liquid State Machine based SNN accelerator in Wang et al. (2016), we redesign and implement the time-compressed Liquid State Machine based SNN on FPGA.

Three speech/image recognition datasets are adopted for benchmarking. The first dataset is a subset of the TI46 speech corpus (Liberman et al., 1991) and consists of 260 isolated spoken English letters recorded by a single speaker. The time domain speech examples are pre-processed by the Lyon's passive ear model (Lyon, 1982) and transformed to 78 channel spike trains using the BSA spike encoding algorithm (Schrauwen and Van Campenhout, 2003). The second one is the CityScape dataset (Cordts et al., 2016) which contains 18 classes of 1,080 images of semantic urban scenes taken in several European cities. Each image is segmented and remapped into a size of 15 ×15, are then converted to 225 Poisson spike trains with the mean firing rate proportional to the corresponding pixel intensity. The third one is a subset of N-TIDIGITS18 speech dataset (Anumula et al., 2018) which is obtained by playing the audio files from the TIDIGITS dataset to a CochleaAMS1b sensor. This dataset contains 10 classes of single digits (the digits “0” to “9”). There are 111 male and 114 female speakers in the dataset and 2,250 training and 2,250 testing examples. For the first two datasets, we adopt 80% examples for training and the remaining 20% for testing. The three datasets present two different types tasks, i.e., speech vs. image classification, and are based on three different raw input encoding schemes, i.e., the BSA encoding, Poisson-based rate coding, and CochleaAMS1b sensor based coding. Therefore, they are well-suited for testing the generality of the proposed time compression.

The baseline LSM FPGA accelerator (without compression) we built in this paper is referred to Wang et al. (2016), which is based on the standard LIF model, and consists of an input layer, a recurrent reservoir, and a readout layer. The number of input neurons is set by the number of the input spike trains, which is 78, 225, and 64, respectively for the TI46 dataset, CityScape dataset, and N-TIDIGITS18 dataset, respectively. The reservoir has 135 neurons for the TI46 and CityScape datasets and 300 neurons for the N-TIDIGITS18 dataset, respectively. Each input neuron is randomly connected to 16 reservoir neurons. The connection probability among two reservoir neurons decays in their Euclidean distance to mimic the connectivity of biological brains (Maass et al., 2002). The number of the readout neurons is 26, 18, and 10 for the TI46, CityScape, and N-TIDIGITS18 dataset, respectively. The reservoir neurons are fully connected to the readout neurons. All readout synapses are plastic and trained using the supervised spike-dependent training algorithm in Zhang et al. (2015). The power consumption of various FPGA accelerators is measured using the Xilinx Power Analyzer (XPA) tool and their recognition performances are measured from the FPGA board. The runtime is the time the proposed FPGA accelerator takes to complete 1 training and testing epoch with an operation frequency of 50 MHz.

### 3.1. Input Spike Train Compression

**Figure 8:**
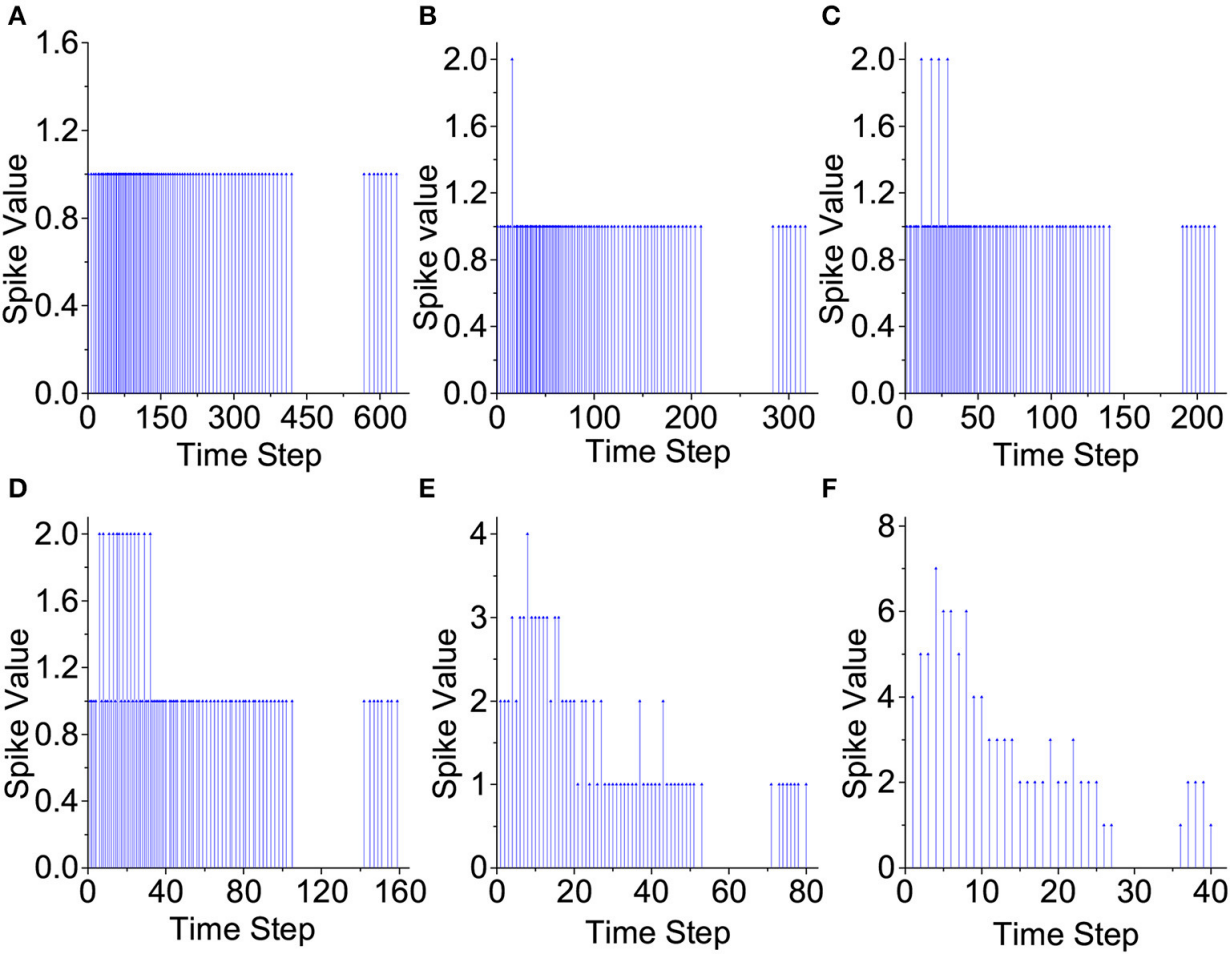
Proposed input compression of a speech example. **(A)** No compression, **(B)** 2:1 compression, **(C)** 3:1 compression, **(D)** 4:1 compression, **(E)** 8:1 compression, and **(F)** 16:1 compression.

Figure 8 demonstrates how the proposed input spike compression unit (ISCU) compresses one input spike train of the spoken English letter “A” from the TI46 speech dataset (Liberman et al., 1991). As in Figure 8, the inter-spike interval of the raw input spike train can be as low as 0 so that any brute-force time compression leads to loss of spikes and hence information loss, jeopardizing classification accuracy. ISCU compresses the raw spikes by converting them to the input-weighted (IW) representation in which densely populated regions of the input train are represented by spikes with a weight greater than one without any spike loss. This makes it possible to dramatically shrink the duration of the spike train while capturing the global temporal distribution of the input spikes using the spike weights. As demonstrated later, ISCU is able to compress the raw input spike trains by large compression ratio while retaining the essential input information necessary for accurate pattern recognition.

### 3.2. Behavior of the Proposed IOW-LIF Neurons

The proposed IOW-LIF neurons play the important role of processing the input-weighted spike trains produced by ISCUs. Except for time compression, the outputs of these IOW-LIF neurons shall be identical or close to the standard LIF neurons receiving the uncompressed spike inputs. We select a single neuron at random from the reservoir of the LSM design for The I46 dataset to observe its membrane potential and the output spike train.

**Figure 9:**
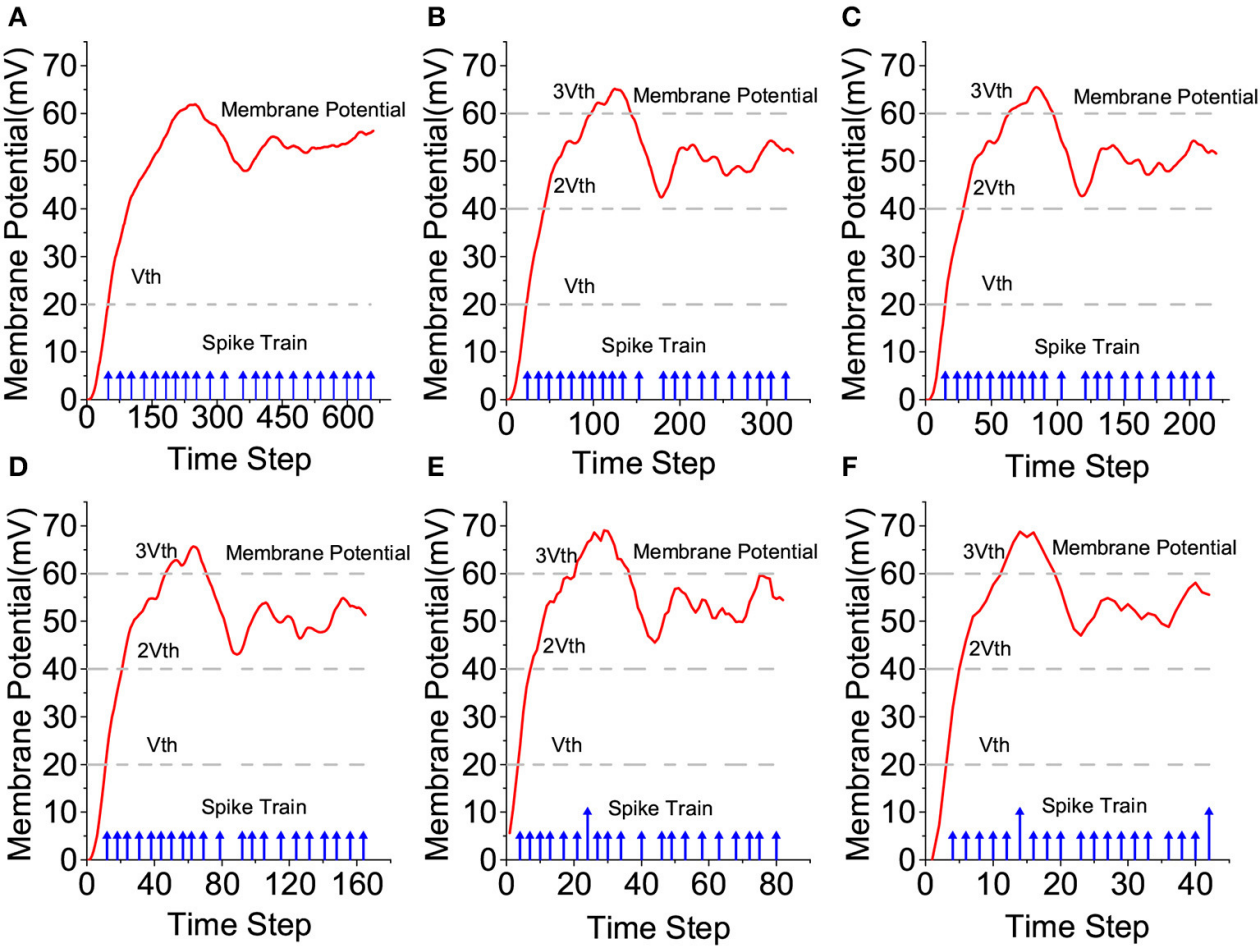
Comparison of LIF and IOW-LIF neurons. **(A)** No compression, **(B)** 2:1 compression, **(C)** 3:1 compression, **(D)** 4:1 compression, **(E)** 8:1 compression, and **(F)** 16:1 compression.

To ease the comparison, the membrane potential is not reset by output firing in Figure 9. It can be seen that the waveform of the membrane potential and output spike train produced by the IOW-LIF neuron bear close resemblance to those of the LIF neuron. It shall be noted that existence of minor difference between the two neurons typically does not lead to large recognition performance drop since such difference is factored in during the training of the SNN, which is based on the same time compression ratio.

### 3.3. Reservoir Responses of the LSMs

**Figure 10:**
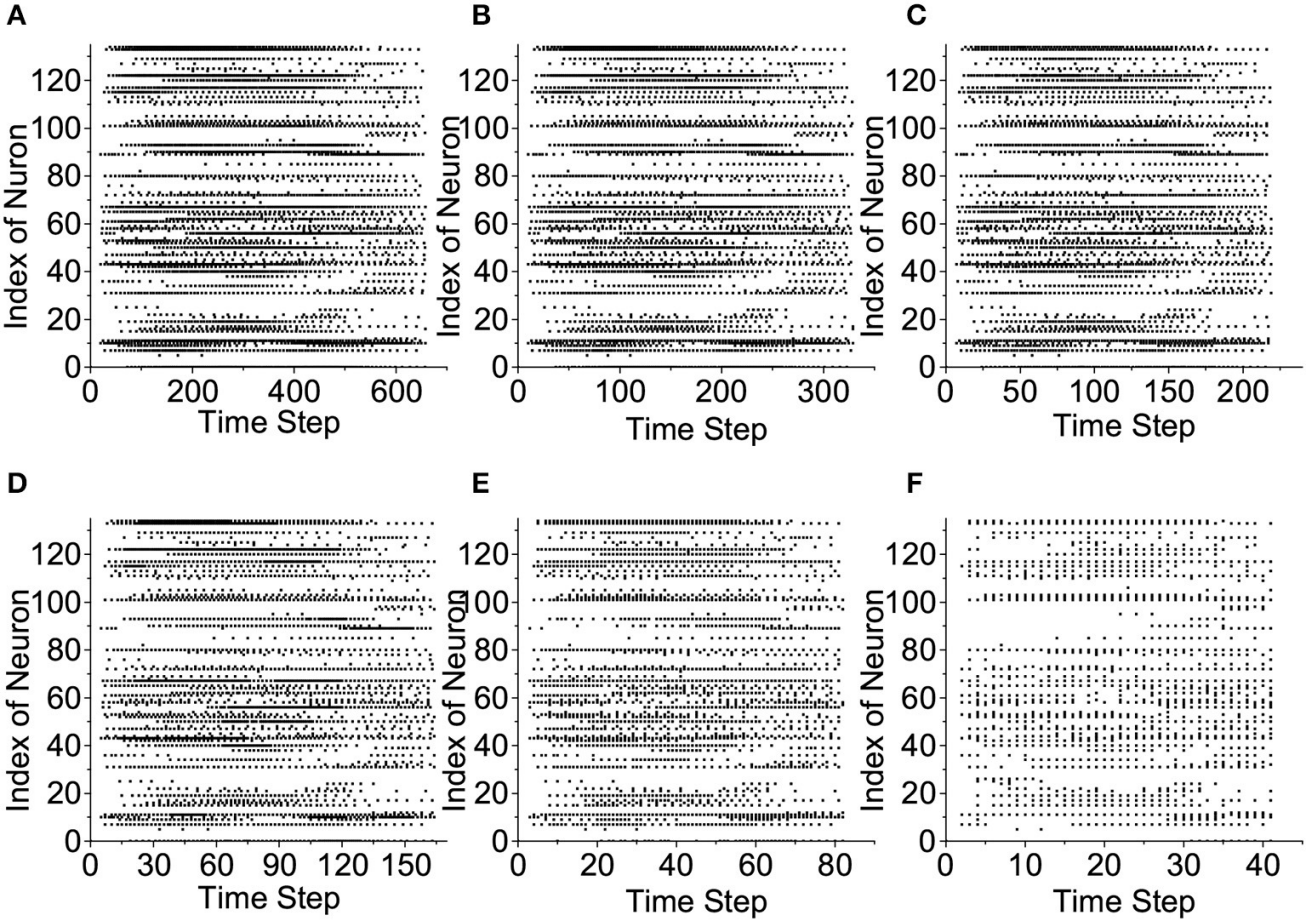
Reservoir response vs. compression ratio. **(A)** No compression, **(B)** 2:1 compression, **(C)** 3:1 compression, **(D)** 4:1 compression, **(E)** 8:1 compression, and **(F)** 16:1 compression.

We plot the raster plots of the reservoir IOW-LIF neurons when the input speech example is the letter “A” from the TI46 Speech Corpus to examine the impact of time compression in Figure 10. It is fascinating to observe that when the compression ratio is between 2:1 to 4:1, the reservoir response in terms of both total spike count and spatio-temporal spike distribution changes little from the one without compression. When the compression ratio increases to the very large values of 8:1 and 16:1, the total spike count drops but the original spatio-temporal spike distribution is still largely preserved. Since certain spike counts are converted to firing spike weights by the IOW neurons, the information of spike count will not be lost. This is consistent to the decent recognition performance achieved at 8:1 and 16:1 compression ratios presented next.

#### 3.3.1. Performances of TC-SNNs With IOW LIF Neurons

For the three datasets mentioned, we design a baseline LSM SNN without time compression and five time-compressed SNNs (TC-SNNs) with IOW LIF neurons referred to Wang et al. (2016) and a fixed time compression ratio from 2:1 to 16:1, all clocked at 50MHz.

For the TI46 speech dataset (Liberman et al., 1991), the runtime and energy dissipation of each accelerator expended on 350 training epochs of a batch of 208 randomly selected examples are measured. We compare the inference accuracy, hardware overhead measured by FPGA lookup (LUT) and flip-flop (FF) utilization, power, runtime, and energy of all six accelerators in Table 1. For the inference accuracy, we measure the best accuracy and average accuracy of multiple experiments with different initial weights and the standard deviation (STD). The same evaluation method is used hereinafter. To show the benefit of producing weighted output spikes, we create a new input-weighted (IW) LIF model which differs from the IOW LIF model in that the IW model generates binary output spikes. We redesign the five TC-SNN accelerators using IW LIF neurons and compare them with their IOW counterparts in Table 1. With large compression ratios the IOW accelerators significantly outperform their IW counterparts on classification accuracy. For example, the IOW accelerator improves accuracy from 69.23 to 80.77% with a compression ratio of 16:1.

**Table 1:** Comparison of the baseline and TC-SNN accelerators with IW/IOW LIF neurons based on TI46 Speech Corpus.

**Compression ratio**	**Neuron model**	**Best accuracy**	**Average accuracy (STD)**	**LUT**	**FF**	**Power (W) @50 MHz**	**Runtime(s) (normalized runtime)**	**Runtime speedup**	**Energy (J) (normalized energy)**	**Energy reduction ratio (%)**	**Normalized ATEL**
Baseline	LIF	96.15%	95.58% (0.89%)	57326	18200	0.073	1.991 (100%)	1.00x	0.145 (100%)	1.00x	100%
2:1	IW-LIF	96.15%	95.29% (0.96%)	58497	18460	0.077	0.995 (49.97%)	2.00x	0.077 (52.71%)	1.88x	26.68%
2:1	IOW-LIF	96.15%	95.50% (0.92%)	60096	18532	0.086	0.995 (49.97%)	2.00x	0.086 (58.87%)	1.69x	30.72%
3:1	IW-LIF	90.38%	89.81% (0.89%)	58538	18549	0.079	0.663 (33.30%)	3.00x	0.052 (35.86%)	2.79x	30.46%
3:1	IOW-LIF	90.38%	89.94% (0.81%)	60103	18625	0.088	0.663 (33.30%)	3.00x	0.058 (40.00%)	2.50x	34.78%
3:1	IW-LIF (TATC)	92.31%	92.08% (0.63%)	58762	18782	0.080	0.664 (33.35%)	3.00x	0.053 (36.55%)	2.74x	24.98%
3:1	IOW-LIF (TATC)	92.31%	92.13% (0.55%)	61162	18799	0.092	0.664 (33.35%)	3.00x	0.061 (42.03%)	2.38x	29.74%
4:1	IW-LIF	92.31%	91.46% (0.96%)	58910	18753	0.081	0.499 (25.06%)	3.99x	0.036 (27.81%)	4.03x	14.31%
4:1	IOW-LIF	92.31%	91.58% (0.80%)	61313	18923	0.095	0.499 (25.06%)	3.99x	0.047 (32.62%)	3.09x	17.40%
8:1	IW-LIF	80.77%	80.44% (0.73%)	59210	19087	0.083	0.248 (12.46%)	8.03x	0.021 (14.16%)	6.90x	9.12%
8:1	IOW-LIF	86.54%	85.87% (0.92%)	62548	19098	0.099	0.248 (12.46%)	8.03x	0.025 (16.89%)	5.80x	7.98%
16:1	IW-LIF	69.23%	68.50% (0.94%)	59400	20000	0.117	0.125 (6.28%)	15.93x	0.015 (10.06%)	9.67x	5.28%
16:1	IOW-LIF	80.77%	80.17% (0.89%)	65349	20808	0.134	0.125 (6.28%)	15.93x	0.017 (11.52%)	8.53x	4.12%

Firstly, we compare the TC-SNN accelerators based on IW-NEs and the TC-SNN accelerators based on IOW-NEs. As Table 1 shows that TC-SNN accelerators based on IW-NEs and TC-SNN accelerators based on IOW-NEs obtain the same accuracy, when the compression ratio is small. However, the accuracy of TC-SNN accelerators based on IW-NEs drop rapidly when compression ratio is large, e.g., the accuracy is only 69.23% when compression ratio is 16:1. While the accuracy of TC-SNN accelerators based on IOW-NEs still is 80.77%. This shows that IOW-NEs can preserve the spike information, effectively, when compression ratio is large.

Secondly, we compare the TC-SNN accelerators using Time Averaging time compression(TATC) and without using time average time compression, when compression ratio is 3:1. As Table 1 shows that TC-SNN using TATC improve the accuracy from 90.38 to 92.31%. Due to the introduction of time average, hardware cost will increase a little. This shows that time average time compression can improve the accuracy when compression ratio is not a power of 2. We will apply time average time compression in our experiments when the compression ratio is not a power of 2.

The power/hardware overhead of the TC-SNN accelerators with IOW LIF neurons only increases modestly with the time compression ratio. For the TC-SNN accelerators, as the compression ratio increase, the throughput steadily improves, reducing the runtime and energy dissipation. Over a very wide range of compression ratio, the runtime is linearly scaled with the compression ratio while the energy is scaled almost linearly. For example, 2:1 compression speeds up the runtime by 2×, reduces the energy by 1.69×, retaining the same classification accuracy of 96.15% without degradation. With 4:1 compression, the runtime is sped up by 3.99×, the energy is reduced by 3.09×, and the classification accuracy is as high as 92.31%. With a large 16:1 compression ratio, the runtime and energy are reduced significantly by 15.93× and 8.53×, respectively, and the accuracy is 80.77%.

To jointly evaluate the tradeoffs between hardware area, runtime, energy, and loss of accuracy, we define a figure of merit (FOM) ATEL as: ATEL = Area × Time × Energy × Loss, where each metric is normalized with respect to the baseline (no compression), and Loss = (100% - Classification Accuracy). Here the hardware area is evaluated by Flop count + 2*LUT count as suggested by Xilinx. Table 1 shows that as the compression ratio increases from 1:1 to 16:1, the ATEL of the TC-SNNs with IOW LIF neurons favorably drops from 100 to 4.12%, a nearly 25-fold reduction.

We evaluate the proposed architectures using the CityScape image recognition dataset (Cordts et al., 2016) and N-TIDIGITS18 dataset (Anumula et al., 2018) in a similar way. The results for the CityScape dataset are reported in Table 2, for which the runtime and energy dissipation of each accelerator are measured for 350 training epochs of a batch of 864 randomly selected examples. Since the proposed compression is application independent, the TC-SNN architectures can be applied to this image recognition task without any modification. Large runtime and energy reductions similar to the ones for the TI46 dataset are achieved by the proposed time compression while the degradation of classification accuracy is more graceful. The TC-SNN with 8:1 compression reduces the runtime and energy dissipation by 7.92× and 6.53×, respectively while the accuracy only drops to 95.37%. The figure of merit ATEL improves from 100 to 2.84% (35× improvement) when the TC-SNN runs with 16:1 compression. The results on the N-TIDIGITS18 dataset are in Table 2, for which the runtime and energy dissipation of each accelerator are measured for 350 training epochs of a batch of 2,250 training samples. Again, large runtime and energy reductions are achieved by the proposed time compression. The TC-SNN with 8:1 compression ratio reduces the runtime and energy dissipation by 7.92× and 5.82×, respectively while the accuracy only drop from 83.63 to 80.91%.

**Table 2:** Comparison of the baseline and TC-SNN accelerators with IOW LIF neurons based on the CityScape image dataset and the NTIDIGITS18 dataset.

**Compression ratio**	**Neuron model**	**Best accuracy**	**Average accuracy (STD)**	**LUT**	**FF**	**Power (W) @50 MHz**	**Runtime(s) (normalized runtime)**	**Runtime speedup**	**Energy (J) (normalized energy)**	**Energy reduction ratio (%)**	**Normalized ATEL**
**CityScape image dataset**											
Baseline	LIF	99.07%	98.94% (0.31%)	57017	16373	0.074	1.497 (100%)	1.00x	0.111 (100%)	1.00x	100%
2:1	IOW-LIF	99.07%	98.75% (0.36%)	58826	17294	0.078	0.749 (50.03%)	2.00x	0.058 (52.25%)	1.91x	27.31%
3:1	IOW-LIF	97.69%	97.53% (0.30%)	58895	17506	0.088	0.499 (33.33%)	3.00x	0.044 (39.64%)	2.52x	34.05%
4:1	IOW-LIF	97.69%	97.39% (0.39%)	59276	17374	0.082	0.375 (25.05%)	3.99x	0.031 27.93%)	3.58x	18.00%
8:1	IOW-LIF	95.37%	95.21% (0.31%)	61254	19322	0.092	0.189 (12.63%)	7.92x	0.017 (15.32%)	6.53x	10.73%
16:1	IOW-LIF	94.91%	94.76% (0.32%)	66350	21618	0.079	0.096 (6.41%)	15.59x	0.008 (7.21%)	13.88x	2.84%
**NTIDIGITS18 dataset**											
Baseline	LIF	83.63%	83.38% (0.29%)	106263	25778	0.116W	424.61 (100%)	1.00x	49.255 (100%)	1.00x	100%
2:1	IWIO-LIF	82.82%	82.50% (0.32%)	111688	26070	0.110W	212.31 (50.00%)	2.00x	23.354 (47.41%)	2.11x	26.04%
3:1	IWIO-LIF	82.22%	81.93% (0.29%)	124756	28364	0.112W	141.50 (33.32%)	3.00x	15.848 (32.18%)	3.11x	13.58%
4:1	IWIO-LIF	81.91%	81.60% (0.29%)	112224	26158	0.113W	106.87 (25.17%)	3.97x	12.076 (24.52%)	4.08x	7.17%
8:1	IWIO-LIF	80.91%	80.56% (0.31%)	131614	28934	0.158W	53.61 (12.63%)	7.92x	8.470 (17.20%)	5.82x	3.10%
16:1	IWIO-LIF	74.54%	74.26% (0.29%)	128094	34707	0.174W	27.17 (6.40%)	15.63x	4.728 (9.60%)	10.42x	1.16%

Clearly, the proposed compression architectures can linearly scale the runtime, and hence dramatically reduce the decision latency, and energy dissipation with acceptable accuracy degradation at low compression ratios, e.g., up to 4:1. Applying an aggressively large compression ratio can produce huge energy and runtime reduction while the degraded performance may be still acceptable for practical applications. The supported large range of compression ratio offers the user great flexibility in targeting an appropriate performance/overhead tradeoff for a given application.

#### 3.3.2. Performances of TC-SNNs With Bursting Coding

We redesign our TC-SNN accelerators using bursting IOW LIF models to support burst coding (Park et al., 2019) and compare their performances with the baseline on the TI46 speech dataset and CityScape image dataset in Table 3. Once again, the proposed time compression leads to large runtime and energy reductions and the degradation of classification accuracy is graceful. The additional hardware cost for supporting bursting coding is somewhat increased but still rather oderate.

**Table 3:** Comparison of the baseline and TC-SNN accelerators with burst coding on the TI46 Speech Corpus and on CityScape image dataset.

**Compression ratio**	**Neuron model**	**Best accuracy**	**Average accuracy (STD)**	**LUT**	**FF**	**Power (W) @50 MHz**	**Runtime(s) (normalized runtime)**	**Runtime speedup**	**Energy (J) (normalized energy)**	**Energy reduction ratio (%)**	**Normalized ATEL**
**TI46 Speech Corpus dataset**											
Baseline	LIF	98.08%	97.42% (0.92%)	92052	62390	0.240W	2.527 (100%)	1.00x	0.606 (100%)	1.00x	100%
2:1	IOW-LIF	92.31%	91.83% (0.84%)	107263	64845	0.163W	1.266 (50.10%)	2.00x	0.206 (33.99%)	2.94x	77.38%
3:1	IOW-LIF	92.31%	91.60% (0.93%)	124881	67343	0.168W	0.946 (37.44%)	2.67x	0.158 (26.07%)	3.82x	50.55%
4:1	IOW-LIF	92.31%	90.56% (1.57%)	102362	61332	0.172W	0.637 (25.21%)	3.97x	0.110 (18.15%)	5.54x	19.68%
8:1	IOW-LIF	88.46%	87.67% (0.95%)	121183	64481	0.212W	0.318 (12.58%)	7.94x	0.067 (11.06%)	9.00x	10.47%
16:1	IOW-LIF	80.77%	79.85% (0.97%)	132055	72508	0.289W	0.163 (6.45%)	15.50x	0.047 (7.76%)	12.87x	6.85%
**CityScape image dataset**											
Baseline	LIF	98.61%	98.42% (0.32%)	92166	60721	0.242	1.899 (100%)	1.00x	0.460 (100%)	1.00x	100%
2:1	IOW-LIF	98.15%	97.58% (0.49%)	106416	63765	0.156	0.950 (50.03%)	1.99x	0.148 (32.25%)	3.10x	24.18%
3:1	IOW-LIF	98.15%	96.80% (0.65%)	123037	66208	0.191	0.633 (33.33%)	3.00x	0.121 (26.31%)	3.80x	14.87%
4:1	IOW-LIF	97.69%	95.83% (1.52%)	100748	59941	0.163	0.476 (25.05%)	3.99x	0.078 (16.87%)	5.93x	7.54%
8:1	IOW-LIF	96.29%	95.03% (0.57%)	120312	64863	0.209	0.239 (12.62%)	7.92x	0.050 (10.90%)	9.17x	4.56%
16:1	IOW-LIF	96.29%	94.89% (1.25%)	133479	73476	0.241	0.122 (6.42%)	15.59x	0.029 (6.38%)	15.66x	1.50%

#### 3.3.3. Performances of Time Compressed Multi-Layer Feedforward SNN With IOW LIF Neurons

To show the generality of our proposed method, we design a pre-trained multi-layer feedforward SNN (196-100-100-10) based on the design in Lee et al. (2019) as the baseline and redesign the pre-trained multi-layer feedforward SNN with time compression for the MNIST dataset (LeCun et al., 1998). Each pixel value of the MNIST image is converted into a spike train using Poisson sampling and the probability of spike generation is proportional to the pixel intensity. Due to the limited hardware resources available, we crop each image to include only 14 ×14 pixels around the center for FPGA evaluation. The spike-train level direct feedback alignment (ST-DFA) algorithm, which is proposed in Lee et al. (2019) , is used to pre-train the multi-layer SNN. The experimental results are shown in Table 4. Compared to the baseline, the pre-trained multi-layer feedforward SNN with 16:1 compression ratio reduces the runtime and energy dissipation by 14.92× and 11.76×, respectively, while the accuracy only drops from 96.48 to 93.01%. As the Table 4 is shown, the additional hardware cost for supporting time compression increases moderately.

**Table 4:** Comparison of the baseline and time compressed multi-layer feedforward SNN accelerators on the MNIST dataset.

**Compression ratio**	**Neuron model**	**Accuracy**	**LUT**	**FF**	**Power (W) @50 MHz**	**Runtime(s) (normalized runtime)**	**Runtime speedup**	**Energy (J) (normalized energy)**	**Energy reduction ratio (%)**	**Normalized ATEL**
baseline	LIF	96.48%	34779	5910	0.376	32.23(100%)	1.00x	12.118(100%)	1.00x	100%
2:1	IOW-LIF	96.09%	38743	6084	0.391	16.35(50.73%)	1.97x	6.392(52.75%)	1.90x	67.48%
3:1	IOW-LIF	95.96%	44213	6227	0.399	10.88(33.76%)	2.96x	4.341(35.82%)	2.89x	54.72%
4:1	IOW-LIF	95.92%	46254	6260	0.416	8.23(25.53%)	3.91x	3.423(28.25%)	3.54x	47.42%
8:1	IOW-LIF	95.58%	47635	6134	0.440	4.18(12.97%)	7.71x	1.839(15.18%)	6.59x	29.97%
16:1	IOW-LIF	93.01%	47955	6140	0.477	2.16(6.70%)	14.92x	1.030(8.50%)	11.76x	28.96%

#### 3.3.4. Performances of PTC-SNNs With Reconfigurable Compression Ratio

We also design a time-compressed SNN (PTC-SNN) accelerator supporting programmable ratio ranging from 2:1 to 16:1 and evaluate it using the TI46 dataset in Table 5. The LUT and FF utilizations of PTC-SNN are 7,4742 and 2,1391, respectively. The overall hardware area overhead stays constant with the programmable compression ratio, which is only 12.78% more than that of the TC-SNN accelerator with a fixed 16:1 compression ratio. Here the hardware area is also evaluated by Flop count + 2*LUT count. The runtime and accuracy of the PTC-SNN are identical to those of the corresponding TC-SNN running on the same (fixed) compression ratio. The energy overhead of the PTC-SNN is still near linearly scaled down by the compression ratio albeit that it is somewhat greater than that of the corresponding TC-SNN. And yet, the PT-SNN reduces the energy dissipation and ATEL of the baseline by 6.59x and 16.53x, respectively when running at 16:1 compression ratio.

**Table 5:** Performances of the reconfigurable PTC-SNN hardware accelerator on the TI46 Speech Corpus.

**Compression ratio**	**Best accuracy**	**Average accuracy (STD)**	**Power (W) @50 MHz**	**Runtime(s)**	**Energy (J)**	**Normalized ATEL**
Baseline	96.15%	95.58%(0.89%)	0.073	1.991	0.145	100%
2:1	96.15%	95.50%(0.92%)	0.151	0.995	0.130	57.64%
3:1	92.31%	89.94%(0.81%)	0.152	0.664	0.088	51.65%
4:1	92.31%	91.58%(0.80%)	0.155	0.499	0.067	29.87%
8:1	86.54%	85.87%(0.92%)	0.173	0.248	0.038	14.61%
16:1	80.77%	80.17%(0.89%)	0.194	0.125	0.022	6.05%

## 4. Discussion

SNNs can support a variety of rate and temporal spike codes among which rate coding using Poisson spike trains has been popular. However, in that case, the low-power advantage of SNNs may be offset to certain extend by long latency during which many spikes are processed for ensuring decision accuracy. This work aims to boost the throughput and reduce energy dissipation of SNN accelerators by temporal compression. We propose a general compression technique that preserves both the spike count and temporal characteristics of the original SNN with low information loss. It transparently compresses duration of the spike trains on top of an existing rate/temporal code to reduce classification latency.

More specifically, the proposed temporal compression aims to preserve the spike counts and temporal behaviors in the neural dynamics, synaptic responses, and dynamics employed in the given SNN such that no substantial alterations are introduced by compression other than that the time-compressed SNN just effectively operates on a faster time scale. However, there are several challenges when we work toward achieving the above goal. Firstly, we propose a new weighted form for representing compressed spike trains, where multiple adjacent binary spikes are compressed into a single weighted spike with a weight value equal to the number of binary spikes combined, allowing preservation of spike information even under very large compression ratios. Furthermore, we proposed a new family of input-output-weighted (IOW) spiking neural models which take the input spike trains in the weighted form and produce the output spike train in the same weighted form, where the multi-bit weight value of each output spike reflects the amplitude of the membrane potential as a multiple of the firing threshold. Finally, we proposed a method to scale the time constant of SNN dynamic to preserve the spike counts and temporal behaviors in the neural dynamics.

In the experimental studies, we propose a general time compression technique and two compression architectures, namely TC-SNN and PTC-SNN, to significantly boost the throughput and reduce energy dissipation of SNN accelerators. Our experimental results show that the proposed time compression architectures can support large time compression ratios of up to 16×, delivering up to 15.93×, 13.88×, and 86.21× improvements in throughput, energy dissipation, and a figure of merit (ATEL), respectively, and be realized with modest additional hardware design overhead on a Xilinx Zynq-7000 FPGA. Our future work will explore the relationship between compression ratio and the information loss. Based on the relationship between them, we can further propose a method to tune the compression ratio, automatically.

## Data Availability Statement

The datasets generated for this study are available on request to the corresponding author.

## Author Contributions

CX and PL developed the theoretical approach for the proposed time compression techniques and wrote the paper. CX and YL implemented the FPGA spiking neural accelerators. CX and WZ performed the simulation studies. CX performed this work at the University of California, Santa Barbara while being a visiting scholar from Xidian University.

### Conflict of Interest

The authors declare that the research was conducted in the absence of any commercial or financial relationships that could be construed as a potential conflict of interest.
